# Influence Mechanism of Social Support of Online Travel Platform on Customer Citizenship Behavior

**DOI:** 10.3389/fpsyg.2022.842138

**Published:** 2022-04-08

**Authors:** Yu-mei Ning, Chuan Hu

**Affiliations:** ^1^School of Business Administration, Zhongnan University of Economics and Law, Wuhan, China; ^2^Business School, Yulin Normal University, Yulin, China

**Keywords:** customer citizenship behavior, social support, online travel platform, consumption emotion, customer satisfaction

## Abstract

Customer citizenship behavior has attracted widespread attention from scholars due to its capacity of enabling enterprises to gain competitive advantages of low costs and high efficiency by giving full play to the initiative of customers. Based on the S-O-R Model, we have established the theoretical model to study the influence imposed by social support of online travel platform enterprises on customer citizenship behavior against the backdrop of the sharing economy. This research tests the fitting of the theoretical model and its fundamental hypotheses using 626 samples acquired from the investigation with SPSS26.0 and AMOS25.0. Results indicate that the social support has a positive influence on customer citizenship behavior. Social support could have a positive influence on positive emotions. Social support has no significant negative effect on negative emotions. Positive emotions have a positive influence on customer citizenship behavior. Negative emotions have a negative influence on customer citizenship behavior. Positive emotions play a mediating effect in the positive influence of social support on customer citizenship behavior. Social support could have a positive influence on customer satisfaction. Customer satisfaction has a positive influence on customer citizenship behavior. Customer satisfaction plays a mediating effect in the positive effect of social support on customer citizenship behavior. Positive emotions and customer satisfaction play a chain mediating effect in the positive effect of social support on customer citizenship behavior.

## Introduction

As the Internet technologies are increasingly popularized and applied, the travel platform enterprises can empower their full-time internal staff to cooperate with customers who have been turned into part-time *external staff* while serving customers, so as to maximize the corporate value. Customers are expected to engage in citizenship behavior in favor of enterprises like full-time internal staff. For example, customers recommend products or services of the travel platform to others in the comments of the online travel platform (e.g., Flying Pig, Qunar), and even customers who have enjoyed the platform will conduct unpaid promotions on social media (such as Douyin, Twitter) in the form of text and video, which taps more potential customers for the online travel platform, thereby expanding the consumer group of the online travel platform. There are also customers who actively provide valuable travel experience feedback to the travel platform, and actively provide information help to other customers who encounter travel problems (Shen and Liu, 2015). The citizenship behavior, which features collaborative creation of value by customers and enterprises, has attracted widespread attention from scholars due to its capacity of enabling enterprises to gain competitive advantages of low costs and high efficiency by giving full play to the initiative of customers. Groth (2005), referring to the definition of organizational citizenship behavior, defined customer citizenship behavior as the beneficial behavior exhibited by customers that are valued and appreciated by firms, and external to the role of customers.

The social support of travel platform enterprises plays an important role in the influence of customer citizenship behavior. With the increasingly popularized application of Internet technologies, the online travel platform enterprises are not only able to provide effective information for customers anytime and anywhere, but also capable of establishing interpersonal relationships with customers and provide them with emotional support such as a sense of belonging, so as to enhance the level of satisfaction and the purchase intention of customers (Chang et al., 2015; Hu et al., 2017). Some of the existing studies have explored the impact imposed by social support on customer citizenship behavior. Originated from the science of rehabilitation psychology and epidemiology, the theoretical concepts of social support have been introduced by a few scholars into their studies on consumer behavior. In such context, the social support refers to the most critical social value that the individual acquires from the enterprise as well as the individual’s perception of available social resources (Chiang and Huang, 2016). Through the empirical analysis of fitness industry, Rosenbaum and Massiah (2007) found that when customers could gain more social support from other customers, they are prone to show higher level of engagement, cooperation and loyalty, and would take a more proactive part in citizenship behaviors such as spreading the word of mouth and assisting other customers. Verleye et al. (2014) argued that the social support that nursing home members receive from nursing home staff and other nursing home members could promote customer citizenship behavior through customer affection (satisfaction) and customer role preparation. Chang et al. (2015) held that the social support that electronics virtual community members receive from other members could enhance the customer citizenship behavior through the intermediary role of relationship quality. Chiu et al. (2015) argued that the social support that online community members of maternal health-related topics received from other members could promote customer citizenship behavior through subjective well-being and community identity. Zhu et al. (2016) held that the social support that electronic virtual community members receive from the community and other members affects customer citizenship behavior through customer satisfaction. Compared with offline traditional service organizations, online platforms are more social and thus provide more opportunities for users to provide social support because they can transcend time and geographical restrictions. Different from online communities, online travel platforms are official platforms established by enterprises to provide users with products or services to purchase, rather than communities established by community members. Such platforms established by enterprises are convenient for managing their social support measures. Social support comes from front-line staff and other users of online travel platform. Different sources of social support may have different influence mechanisms on customer citizenship behavior. In addition, when customers receive the service experience of online travel companies that provide users with products or services to purchase, they will inevitably trigger emotional reactions (Richins, 1997). Kim et al. (2013) also stated that the tourists’ emotions may be heavily influenced by interactions with friends and others *via* social media. Moreover, studies have found that emotional state and experience are important factors affecting tourists’ attitudes and behaviors (Su and Hsu, 2013; Su et al., 2016). Emotions have been identified as a key customer resource in the value creation process (Arnould et al., 2006). At present, most scholars adopt the S-O-R Model proposed by Mehrabian and Russell (1974) in their analysis of the formation mechanism of customer citizenship behavior. The S-O-R model suggests that the stimulus of environment could drive individual behavior (Mehrabian and Russell, 1974) through the intermediary role of psychological response, which includes affective and cognitive states (Kim and Lennon, 2013). Therefore, in the process of interacting with enterprises, customers will not only coordinate the relationship between individual behaviors and environmental stimuli through cognitive activities, but also incorporate complex emotional factors. What affects behavioral intention is that emotional factors even have more influence on positive word of mouth, conversion behavior, willingness to pay, and behavioral intentions than cognitive factors (Yu and Dean, 2001). However, most of the existing studies on the influence of social support on customer citizenship behavior haved focuses on the role of cognitive factors such as customer satisfaction (Verleye et al., 2014; Zhu et al., 2016) and social identity (Chiu et al., 2015), ignoring the psychological mechanism of emotional factors in the process of customer consumption experience. Therefore, integrating cognitive factors and emotional factors can more comprehensively answer customers’ citizenship behaviors on online travel platforms. Taking the online travel platform that is easy to provide social support for users and easy to control the social support strategy provided as the research object, based on the S-O-R Model, we have introduced the intermediary variables of consumption emotion (emotional factor) and customer satisfaction (cognitive factor) to explore the influence mechanism of social support on customer citizenship behavior, so as to shed light on ways of enhancing the customer citizenship behavior, service support for online travel platform enterprises and service enterprises.

The potential contribution of this study is firstly from the perspective of research. On the basis of the existing research route of “social support - customer satisfaction - customer citizenship behavior,” we introduce the customer’s emotional factor, and emphasize the importance of emotional responses in social support promoting customer citizenship behavior. Taking the S-O-R model as the logical framework to further reveals the psychological mechanism of customer emotional response and customer cognitive evaluation that social support affects customer citizenship behavior, thereby providing a new perspective for understanding how emotional factors and cognitive factors jointly shape customer citizenship behavior after receiving social support from online travel platforms. Secondly, on the research object, based on online travel platform, this study has explored the influence mechanism of social support on customer citizenship behavior to comprehensively answer how social support from front-line employees of online travel platform and other users of online travel platforms affect customer citizenship behavior.

## Literature Review

### Customer Citizenship Behavior

Customer citizenship behavior refers to the beneficial behavior exhibited by customers that are valued and appreciated by firms, and external to the role of customers (Groth, 2005). Groth (2005) divided the dimensions of customer citizenship behavior into recommendation intention, possibility of providing feedback to the organization and willingness to help other customers. These dimensions have been widely accepted by other scholars (Anaza, 2014). These three dimensions are also used in this study. At present, the majority of scholars elaborate on the emergence of customer citizenship behavior in retail, leisure tourism, virtual brand community and other industries from the perspective of customers’ perception of fairness (Yi and Gong, 2008) and support (Yin and Zhang, 2020), customer satisfaction (Assiouras et al., 2019), and customer engagement (Fan et al., 2015), among other related variables.

### Social Support

Social support refers to the provision of intangible information or emotional support as well as tangible resources or material support for individuals (Cohen and Wilis, 1985). In the network environment, enterprises provide social support to customers mainly through immaterial resources. Therefore, most scholars drew inspiration from the opinions put forward by Schaeferc (1981) and divided the social support of Internet enterprises into informational and emotional value support. Informational value support refers to the information provided in the form of proposal, advice or knowledge that facilitates the problem-solving, whereas emotional value support refers to the provision of emotion-related information, including but not limited to care, understanding, or empathy. Compared with informational value support, emotional value support facilitates customers’ problem-solving from the psychological perspective in an indirect manner. Most study on online social support currently focus on the area of online patient communities (Coulson, 2005).

### S-O-R Model

The S-O-R model suggests that the stimulus of environment could drive individual behavior (Mehrabian and Russell, 1974) through the intermediary role of psychological response, which includes affective and cognitive states (Kim and Lennon, 2013). Therefore, the social support provided by front-line employees and other users of online travel platforms can be regarded as an environmental stimulus, which drives customer citizenship behavior through positive mediating effect on customer psychological response (including consumer emotion and customer satisfaction).

## Theoretical Model and Basic Hypothesis

### Social Support and Customer Citizenship Behavior

Bettencourt (1997), Rosenbaum and Massiah (2007) confirmed that support from enterprise organizations and support from other customers would affect customer recommendation, word-of-mouth, repurchase, cooperative production, suggestion and feedback and other behaviors. Crocker and Canevello (2008) showed that the perception of support from others would lead individuals to reciprocate with similar supportive behaviors. Molinillo et al. (2020) found that social support of brand social commerce websites would affect customers’ intention to create, stickiness, word-of-mouth and repurchase. According to the core principle of social exchange theory, the process of customer consumption experience can be regarded as the process of social exchange. When customers think that enterprises provide good social support in the process of consumption, they will tend to show positive behaviors that are not required to be reciprocated. Therefore, we have put forward Hypothesis H_1:_ Social support can positively affect customer citizenship behavior.

### The Mediating Effect of Positive Emotions and Negative Emotions in Consumption Emotion

Most studies consent to the consumption emotion defined by Mano and Oliver (1993), which refers to a series of emotional responses to the evaluation of product and service value as well as attribute evaluation during consumption experience. In the studies on customer behavior, there is no distinction among consumption emotion, sentiment and feelings. Most scholars divide consumption emotion into two dimensions: positive emotions and negative emotions. Customers may experience a variety of emotions in the process of consumption. Westbrook (1987) pointed out that positive and negative emotions are independent of each other, that is, in a service experience, customers may experience both positive emotions, such as excitement, joy and joy, and negative emotions, such as sadness, annoyance and anger.

Price et al. (1995) found that service skills, sincere attitude, extra care for customers’ interests, mutual understanding between service staff and customers, and polite hospitality behavior of service staff all enhance customers’ positive emotions. Social support from others would bring good emotions to the customer without negative emotions toward the product involved (Zhu et al., 2016). Oh et al. (2014) held that supportive interactions on social networking sites are positively correlated with positive emotions, but have no significant relationship with negative emotions. Chung et al. (2017) found that tourists’ perception of social support on social networking sites can stimulate positive emotions. Rosenbaum (2008) advocated that adequate social support would effectively alleviate customers’ negative emotions such as loneliness, solitude, stress, tension and depression.

According to the theory of emotional evaluation, emotions are generated from individuals’ subjective cognitive evaluation of external environmental stimuli. Once individuals perceive the external environment, they will evaluate the external stimulus information in a meaningful direction for themselves (Arnold, 1960). When service staff provide customers with suggestions to solve the problem or to show customers care, encouragement and other emotions in dealing with problems, customers will make cognitive evaluation of external stimulus information brought by service staff. Good social support evaluation of service staff will usually make customers accompanied by excitement, surprise, happiness, relaxation and other positive emotional experience. With the continuous stimulation of external environmental information of service staff, such positive and meaningful cognitive evaluation is constantly strengthened, and the positive emotional experience of customers is constantly accumulated and enhanced. The negative social support of service staff will make customers with anger, disappointment, regret and other negative emotional experience. Therefore, we have put forward Hypothesis H_2a_: Social support can positively affect positive emotions; H_2b_: Social support can negatively affect negative emotions.

Suh and Kim (2002) found that in online stores, negative emotions have a negative impact on repeat purchase intention, but positive emotions have no significant impact. Zhao et al. (2018) held that positive and negative emotions have a significant impact on customer engagement behavior. Yi and Gong (2006) held that both positive and negative emotions could impose a significant impact on customer citizenship behavior. Bernard et al. (1998) found that when people experience something and have some strong emotions, such as joy, sadness, anger, etc., they tend to share the experience or the emotions with others around them, so as to experience the positive emotions for a long time or relieve the negative emotions. In the process of consumption experience, when consumers have strong positive emotions such as excitement and joy, they will carry out word-of-mouth release or cooperative production for the motivation of sharing joy more easily and rewarding merchants. And vice versa. After assuming that environmental stimuli can trigger individual emotional responses, the theory of emotional evaluation further points out that emotions can induce specific behaviors (Arnold, 1960). Therefore, customers’ behavior after consumption experience is an emotional response to the social support provided by service staff. Therefore, we have put forward Hypothesis H_3a_: Positive emotions can positively affect customer citizenship behavior; H_3b_: Negative emotions can negatively affect customer citizenship behavior.

Jain and Jain (2005) pointed out that the establishment of an optimal relationship between enterprises and customers could facilitate the emotional input of customers, thus encouraging customers to engage in numerous sorts of positive behaviors that are either required or non-mandatory. According to the theory of emotional evaluation, customers’ cognitive evaluation of the external stimulus information brought by the social support of online travel platform will bring about customers’ consumption emotional experience, thus inducing the generation of specific behaviors. Therefore, combining H_2a_, H_2b_, H_3a_, H_3b_, we have put forward Hypothesis H_4a_: Positive emotions can play a mediating effect between social support and customer citizenship behavior, that is, social support will increase customers’ positive emotions so as to increase customer citizenship behavior; H_4b_: Negative emotions can play a mediating effect between social support and customer citizenship behavior, that is, social support will reduce customers’ negative emotions so as to increase customer citizenship behavior.

### The Mediating Effect of Customer Satisfaction

Fornell et al. (1996) defined the customer satisfaction as the subjective assessment made by customers who compare their objective cognition of the consumption results with the expectation of the consumption subsequent to their consumption experience. It has been widely established that social support affects customer satisfaction. Kim and Tussyadiah (2013) found that receiving and perceived social support have a positive impact on travel experience satisfaction. Verleye et al. (2014); Chang et al. (2015), Chiu et al. (2015), and Zhu et al. (2016) found that customers tend to be more satisfied when they could obtain more social support. Suggestions to provide problems or psychological support for customers is part of the enterprise serving customers, and a large number of studies have proved that perceived service quality is significantly positively correlated with satisfaction (Omar et al., 2016). Perceived service quality refers to consumers’ overall views on the quality of service provided by an enterprise. Omar et al. (2016) divided service quality into tangibility, reliability, responsiveness, credibility and empathy, and confirmed the important role of these five dimensions in explaining customer satisfaction. When enterprises provide customers with more reliable suggestions to solve problems, faster and more credible responses, or provide customers with psychological and spiritual support, consumers will be more satisfied with enterprises. Therefore, we have put forward Hypothesis H_5_: Social support can positively affect customer satisfaction.

It has been widely verified that customer satisfaction constitutes a vital factor influencing the customer citizenship behavior (Assiouras et al., 2019). Customer satisfaction is an important quality of the relationship between variables, improve customer satisfaction to strengthen customer relationships with companies. Customers in the consumer and accept the service process, the enterprises provide satisfactory even than expected benefit of goods or services, then increase the level of enterprise relationship quality to customers tend to show is not asked to positive behavior. Therefore, we have put forward Hypothesis H_6_: Customer satisfaction can positively affect customer citizenship behavior.

Jung and Yoo (2017) proposed that customer interaction in entertainment centers promotes customer citizenship behavior through customer satisfaction. Zhao et al. (2014) based on the service context, and examined the influence of social support on customer participation behavior and customer citizenship behavior through relationship commitment. Verleye et al. (2014); Chang et al. (2015), and Zhu et al. (2016) verified that social support promotes customer citizenship behavior through customer satisfaction. According to the S-O-R model, as an external stimulus, social support of online travel platform can effectively improve customer satisfaction and drive customer citizenship behavior through positive mediating effects on both physical and psychological well-being of customers. Therefore, combining H_5_, H_6_, we have put forward Hypothesis H_7_: Customer satisfaction can play a mediating effect between social support and customer citizenship behavior, that is, social support will increase customer satisfaction, thus increasing customer citizenship behavior.

### The Chain Mediating Effect Between Positive Emotions and Negative Emotions and Customer Satisfaction

According to the emotion evaluation theory, when enterprises provide customers with more reliable suggestions to solve problems, faster and more credible responses, or provide customers with more psychological and spiritual support, customers are more likely to make positive cognitive evaluation, and then more likely to stimulate customers’ positive emotions. With the continuous stimulation of service staff’s external environmental information, customers’ positive emotional experience is continuously accumulated and enhanced. The more situational positive emotions accumulated, the easier it is for consumers to form cognitive satisfaction evaluation of enterprises. When customers give high evaluation to the enterprise for providing satisfactory or even exceeding expected goods or services, the quality level of the relationship between customers and the enterprise will be improved, so that customers tend to show positive behaviors that are not required. And vice versa. Although previous literatures have not directly examined the chain mediating effect of consumption emotion and customer satisfaction on the impact of social support on customer citizenship, relevant literatures have verified the mediating effect of customer satisfaction on social support and customer citizenship behavior in the context of virtual brand community (Zhu et al., 2016). Therefore, we have put forward Hypothesis H_8a_: Positive emotions and customer satisfaction can play a chain mediating effect between social support and customer citizenship behavior, that is, social support increases customer satisfaction by increasing customer positive emotions, thus promoting customer citizenship behavior; H_8b_: Negative emotions and customer satisfaction can play a chain mediating effect between social support and customer citizenship behavior, that is, social support can increase customer satisfaction by reducing customer negative emotions, thus promoting customer citizenship behavior.

**FIGURE 1:**
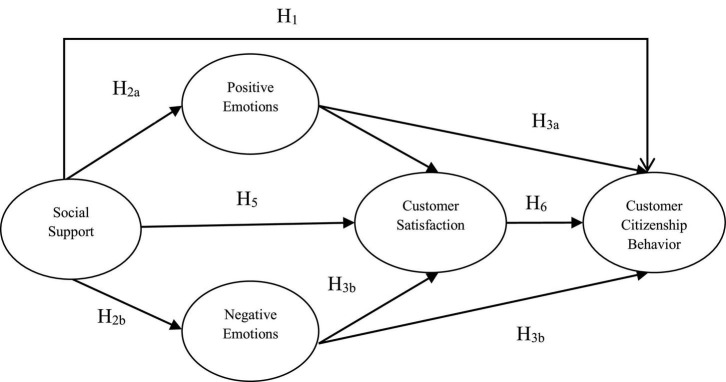
Theoretical model on the impact of social support on customer citizenship behavior.

Therefore, the theoretical model of this study is formed based on the S-O-R model and the above research assumptions, as shown in Figure 1.

## Research Method

### Design of the Questionnaire

As the research context of this study is online travel platform, the scales of this study are designed with online travel platform as the carrier. China’s online travel platforms include Ctrip, Flying Pig and Qunar, which are popular with the younger generation. In addition to the questions for collecting the basic information on the respondents, we have designed 28 questions in this questionnaire (as shown in Table 1). Based on the scale of Chang et al. (2015), the social support can be divided into informational and emotional value support, and we have correspondingly set questions X_ss1_-X_ss8_. Based on the scale of He and Wang (2006), the consumption emotion can be divided into positive emotions and negative emotions, and we have correspondingly set questions X_CE1_-X_CE6_. Based on the scale of Anderson and Fornell (1994), the customer satisfaction can be divided into general satisfaction, expected satisfaction and overall satisfaction, and we have correspondingly set questions X_CS1–_X_CS3_. Based on the scale of Markus Groth (2005), customer citizenship behavior can be divided into recommendation, assistance and feedback, and we have correspondingly set questions X_CC1_-X_CC11_. During the design of questions in the questionnaire, we have adopted the 7-point Likert Scale.

**TABLE 1:** Questionnaire items.

Variable	Item
X_SS1_	The employees or members of the travel platform offered me suggestions to solve the problem.
X_SS2_	The employees or members of the travel platform gave me information to help me overcome the problem.
X_SS3_	The employees or members of this travel platform helped me discover the course and provided me with suggestions to solve the problem.
X_SS4_	The employees or members of this travel platform told me the way to solve the problem.
X_SS5_	The employees or members of this travel platform were on my side with me to face the difficulty.
X_SS6_	The employees or members of this travel platform comforted and encouraged me to face the difficulty.
X_SS7_	The employees or members of this travel platform listened to me talk about my private feelings about the difficulty.
X_SS8_	The employees or members of this travel platform expressed interest and concern in my well-being.
X_CE1_	I feel excited about the service experience provided by this travel platform.
X_CE2_	I feel happy about the service experience provided by this travel platform.
X_CE3_	I feel relaxed about the service experience provided by this travel platform.
X_CE4_	I feel angry about the service experience provided by this travel platform.
X_CE5_	I feel disappointed about the service experience provided by this travel platform.
X_CE6_	I feel regret about the service experience provided by this travel platform.
X_CS1_	Overall, I am satisfied with the service provided by this travel platform.
X_CS2_	Compared with expectations, I am satisfied with the services provided by this travel platform.
X_CS3_	Compared with the ideal situation, I am satisfied with the services provided by this travel platform.
X_CC1_	I would like to recommend this travel platform to my fellow students or coworkers.
X_CC2_	I would like to recommend this travel platform to my family.
X_CC3_	I would like to recommend this travel platform to my peers.
X_CC4_	I would like to recommend this travel platform to people interested in the travel platform’s products or services.
X_CC5_	I would like to introduce this travel platform’s products or services to others.
X_CC6_	I am willing to be a consultant to others and help him choose the goods or services provided by this travel platform.
X_CC7_	I am willing to tell other customers what they should pay attention to when purchasing products on this travel platform.
X_CC8_	I am willing to fill out the customer satisfaction survey of this travel platform.
X_CC9_	I am willing to provide helpful feedback to customer service.
X_CC10_	I am willing to provide information when survey by this travel platform.
X_CC11_	I am willing to inform this travel platform about the great service received by an individual employee.

### Distribution and Recovery of Questionnaires

In this study, we have tested the reliability and validity of the pre-survey data. Both the reliability and the structural validity of each measured variable were deemed as satisfactory, and we have formed the final formal questionnaire accordingly. The research context of this study is online tourism platform, so network survey is applicable. We adopted the non-probabilistic convenience sampling method to collect data from members who have used the online travel platform in recent year through professional online survey websites. After data collection, we conducted strict screening and deleted the questionnaires with obvious regularity and short answer time. A total of 626 valid questionnaires were collected. In terms of geographic regions of respondents (based on IP addresses of respondents), respondents came from 34 provincial administrative regions in China, indicating a large geographic coverage of the sample. In terms of the most commonly used online travel platforms, 30.8% of respondents chose Ctrip, 21.5% chose Flying Pig and 20.7% chose Qunar, indicating that the sample covers major online travel platforms.

### Common Method Deviation Analysis

In this study, two methods were used to test common method bias. First, Harman’s single factor test method was adopted, and exploratory factor analysis method was used to add five variables into a total of 28 items. The results of unrotated factor showed that the total variance of explanation of the first factor was 30.99%, less than 40%, indicating that the problem of common method deviation of data was not serious (Podsakoff et al., 2003). The second method is to test the correlation coefficient between latent variables. If the correlation coefficient between latent variables is less than 0.9, it indicates that the variation degree of common method is acceptable. The correlation coefficient between the latent variables in this study is between 0.24 and 0.73, again indicating that the common method bias problem is not serious (Zhou et al., 2014).

### Reliability and Validity Tests of the Questionnaire

We have calculated the value of Cronbach α of 626 valid questionnaires by using the SPSS26.0 software. The value amounted to 0.91, indicating high reliability of these questionnaires. Furthermore, we have calculated the values of Cronbach α of the five latent variables, namely, social support, positive emotions, negative emotions, customer satisfaction and customer citizenship behavior. The values amounted to 0.88, 0.81, 0.87, 0.87 and 0.82, respectively (as shown in Table 2), indicating good reliability.

**TABLE 2:** Test indicators and fitting results of the measurement model.

Latent variable	Measured variable	Cronbach α	Composite Reliability	Factor Loading
Social support →	X_SS1_			0.56
Social support→	X_SS2_			0.67
Social support→	X_SS3_			0.65
Social support→	X_SS4_			0.81
Social support→	X_SS5_			0.68
Social support→	X_SS6_	0.88	0.90	0.91
Social support→	X_SS7_			0.83
Social support→	X_SS8_			0.63
Positive emotions→	X_CE1_			0.69
Positive emotions→	X_CE2_	0.81	0.82	0.88
Positive emotions→	X_CE3_			0.75
Negative emotions→	X_CE4_			0.89
Negative emotions→	X_CE5_	0.87	0.89	0.86
Negative emotions→	X_CE6_			0.80
Customer satisfaction→	X_CS1_			0.75
Customer satisfaction→	X_CS2_	0.87	0.84	0.80
Customer satisfaction→	X_CS3_			0.85
Customer citizenship behavior→	X_CC1_			0.84
Customer citizenship behavior→	X_CC2_			0.77
Customer citizenship behavior→	X_CC3_			0.74
Customer citizenship behavior→	X_CC4_			0.89
Customer citizenship behavior→	X_CC5_	0.82	0.92	0.71
Customer citizenship behavior→	X_CC6_			0.70
Customer citizenship behavior→	X_CC7_			0.53
Customer citizenship behavior→	X_CC8_			0.67
Customer citizenship behavior→	X_CC9_			0.60
Customer citizenship behavior→	X_CC10_			0.65
Customer citizenship behavior→	X_CC11_			0.69

In this study, we have adopted the AMOS25.0 software to test the convergent validity. The factor loading of all items exceeds the reference value of 0.5 (as shown in Table 2), the composite reliability (CR) values of the five latent variables are higher than 0.8 (as shown in Table 2), and AVE values of the five latent variables are all above 0.5, indicating high level of convergent validity of the aforementioned five latent variables. Meanwhile, the AVE square root value of each latent variable is compared horizontally with the correlation coefficient matrix of other latent variables. The AVE square root values of each latent variable are above their correlation coefficients with other latent variables (as shown in Table 3). As shown in Table 2 and Table 3, the scale of this study features good convergent and discriminant validity.

**TABLE 3:** Differential validity test of latent variables.

	Social support	Positive emotions	Negative emotions	Customer satisfaction	Customer citizenship behavior
Social support	**0.73**				
Positive emotions	0.62	**0.78**			
Negative emotions	0.24	0.35	**0.85**		
Customer satisfaction	0.61	0.70	0.29	**0.80**	
Customer citizenship behavior	0.70	0.62	0.37	0.73	**0.71**

*The data marked in bold in the diagonal line of the matrix is the AVE square root, whereas the rest of the data is the corresponding correlation coefficient.*

### Model Fitting and Hypothesis Testing

In this study, we have fitted the model by using the AMOS25.0 software, and the test results of the theoretical model fitting are specified in Table 4. The CMIN/DF amounted to 2.80. The RMSEA amounted to 0.06, which was deemed as good as it fell within the range between 0.05 and 0.08. With the exception of the GFI, which was slightly equal to 0.9, the CFI, IFI, and TLI all exceeded 0.9. Therefore, the fitting results of the theoretical model and the survey data were deemed as ideal.

**TABLE 4:** Test of theoretical model fitting.

Fitness	X^2^/df	RMSEA	GFI	CFI	IFI	TLI
Index value	2.80	0.06	0.90	0.93	0.93	0.92

As shown by the test results of the hypothesis of model fitting (Table 5), social support of online travel platform could impose a significant positively impact on customer citizenship behavior (Hypothesis **H_1_**). The Hypothesis **H_2b_** is not significant at *P* < 0.001 (^***^) significance level, in other words, the Hypothesis **H_2b_** failed to pass the test; social support of online travel platform could impose a significant positively impact on positive emotions (Hypothesis **H_2a_**). This conclusion is consistent with that of Oh et al. (2014). Social support of online travel platform had a significant positively impact on customer satisfaction (Hypothesis **H_5_**). This conclusion is consistent with that of Zhu et al. (2016). Positive emotions could impose a significant positively impact on customer citizenship behavior (Hypothesis **H_3a_**); Negative emotions could impose a significant negatively impact on customer citizenship behavior (Hypothesis **H_3b_**). This conclusion is consistent with that of Zhao et al. (2018). Customer satisfaction could impose a significant positively impact on customer citizenship behavior (Hypothesis **H_6_**). This conclusion is consistent with that of Zhu et al. (2016).

**TABLE 5:** Test of basic hypothesis.

Hypothesis	Std. Estimate	P	Conclusion
H_1_: Social support → customer citizenship behavior	0.25	***	support
H_2a_: Social support → positive emotions	0.60	***	support
H_2b_: Social support → negative emotions	−0.06	0.193	Non-supportive
H_3a_: Positive emotions → customer citizenship behavior	0.23	***	support
H_3b_: Negative emotions → customer citizenship behavior	−0.13	***	support
H_5_: Social support → customer satisfaction	0.48	***	support
H_6_: Customer satisfaction → customer citizenship behavior	0.65	***	support

Significance levels: P < 0.001 (indicated by***), P < 0.01 (indicated by**), P < 0.05 (indicated by*).

In order to further verify the mediating effects of positive emotions, negative emotions, customer satisfaction and the chain mediating effects of positive emotions, negative emotions and customer satisfaction, the Bootstrap method was used to test the mediating effects in the structural equation model. The results of Bootstrap test are shown in Table 6. After controlling for gender, age, education background, income and other four variables: (1) the indirect effect of social support on customer citizenship behavior through positive emotion is 0.14, and the 95% confidence interval of Bootstrap = 5000 is [0. 005,0. 271], excluding 0 and p < 0.001, that is, hypothesis **H_4a_** pass the test; The indirect effect of social support on customer citizenship behavior through negative emotions is 0.001, and the 95% confidence interval of Bootstrap = 5000 is [−0.012, 0.161], including 0. Therefore, the indirect effect is not significant, that is, hypothesis **H_4b_** failed the test. (2) the indirect effect of social support from customer satisfaction to customer citizenship behavior is 0.31, and the 95% confidence interval of Bootstrap = 5000 is [0. 142,0. 474], excluding 0 and p < 0.001, that is, hypothesis **H_7_** pass the test, this conclusion is consistent with that of Zhu et al. (2016). (3) the chain mediating effect between positive emotions and customer satisfaction on social support and customer citizenship behavior is significant, and the point estimate is 0.15, and the 95% confidence interval of Bootstrap = 5000 is [0. 068,0. 238], excluding 0 and p < 0.001, that is, hypothesis **H_8a_** pass the test; The chain mediating effect of negative emotions and customer satisfaction between social support and customer citizenship behavior is significant, and its point estimate value is 0.01. The 95% confidence interval of Bootstrap = 5,000 is [−0.190, 0.200], including 0. Therefore, the indirect effect is not significant, that is, hypothesis **H_8b_** failed the test.

**TABLE 6:** Mediating effect and 95% confidence interval estimated by Bootstrap method.

The serial number	Concrete indirect effect path decomposition	Indirect effect estimation (Standardization)	Bias-corrected 95%CI	
			Lower	Upper
(1)	SS → PE→ CCB	0.14	0.005	0.271
	SS → NE → CCB	0.001	−0.012	0.161
(2)	SS → CS→ CCB	0.31	0.142	0.474
(3)	SS → PE→ CS→ CCB	0.15	0.068	0.238
	SS → NE →CS → CCB	0.01	−0.190	0.200

## Research Conclusion and Practical Implications

### Research Conclusion

(1) Social support could impose a significant impact on positive emotions instead of the negative emotions. The results indicated that the increased level of social support could enhance the positive emotions, though it failed to reduce negative emotions. The reason behind is that customers believe that the social support constitutes a value added provided by enterprises to them instead of a resource that must be offered. If the level of social support that the enterprises could provide or have provided is utterly high, it is rather easy to stimulate the positive emotions of customers. On the contrary, if the enterprises have provided limited or even no social support at all, customers would not generate negative emotions such as anger, disappointment and regret, etc. Therefore, the social support serves as an incentive rather than a hygiene factor for customers.

(2) The influence coefficient of positive emotions and negative emotions on customer citizenship behavior amounted to 0.23 and −0.13, respectively. The results have shown that the positive emotions are vital for promoting the customer citizenship behavior, whereas the negative emotions are harmful for restraining the customer citizenship behavior.

### Theoretical Significance

(1) This research has expanded the studies on social support in the context of tourism services and enriched the studies on the influence factors of customer citizenship behavior. At present, the research on customer citizenship behavior mainly focuses on the antecedent variables, including but not limited to customer experience (Kim and Choi, 2016) and customer justice perception (Yi and Gong, 2008) in retail and virtual community. Existing studies on the impact of social support on customer citizenship behavior mainly focus on the social support of offline traditional service organizations (Verleye et al., 2014) and online communities (Chiu et al., 2015; Zhu et al., 2016). However, the prevalence of social support in online travel platforms is obvious, and every day, a large number of users of online travel platforms share their experiences of places they have recently visited and exchange travel experiences with other users on the platform (Kim and Tussyadiah, 2013). On the other hand, because the online platform can transcend the limitations of time and space, it is more convenient for users of the platform to obtain social support for tourism services, so that online platforms play an important role in providing social support to users. However, there are still few studies on the social support of online travel platforms. Based on the online travel platform, this research studies the influence mechanism of social support on customer citizenship behavior, and finds the indirect driving role of positive emotions and customer satisfaction in the impact of social support on customer citizenship behavior. These results have provided a comprehensive answer to how social support from front-line employees and other users of online travel platforms affects customer citizenship behavior. which helps extend the existing theories on customer citizenship behavior, which thus enrich the research context of social support and the existing research on the influence factors of customer citizenship behavior.

(2) This research has deepened the internal mechanism of the influence of social support on customer citizenship behavior. Most of the existing studies on the impact of social support on customer citizenship behavior have been focused on the perspective of cognitive factors such as customer satisfaction (Verleye et al., 2014; Zhu et al., 2016), social identity (Chiu et al., 2015) and other cognitive factors, ignoring the role of emotional factors, which also exert a powerful influence on consumer behavior (Ping, 2013). This research takes the S-O-R model as the logical framework, introduces two mediating variables of customer satisfaction and consumption emotion, and comprehensively discusses the internal mechanism of social support on customer citizenship behavior from the perspectives of cognition and emotion. And thus our study has responded to the call to further explore other psychological variables of social support influencing customer citizenship behavior (Zhu et al., 2016) and improved the research on the psychological mechanism of social support influencing customer citizenship behavior.

(3) In this research, we have expanded the studies on the influence factors and influence effects of consumption emotion. At present, related studies on consumption emotion mainly focuses on outcome variables such as customer satisfaction (Lee et al., 2009; Wei and Lin, 2020), customer loyalty (Ou and Verhoef, 2017), and service quality (Lo et al., 2015), shopping environment (Hanzaee and Javanbakht, 2013) and other antecedent variables, few studies have explored the impact of social support on consumption emotion. In addition, most scholars tend to pay more attention to the influence imposed by positive emotions rather than negative emotions on customer behavior (Ma et al., 2017; Behzad et al., 2019). In this study, we have analyzed the influence imposed by social support on consumption emotion, including both positive emotions and negative emotions. Moreover, we have tested the indirect influence imposed by consumption emotion and customer satisfaction on the relationship between social support and customer citizenship behavior. These findings are useful supplements to the existing studies on the influence factors and influence effects of consumption emotion.

### Practical Implications for Management

The influence imposed by the social support offered by online travel platform enterprises on customer citizenship behavior could provide critical inspirations for these firms and the related service enterprises to formulate marketing strategies.

(1) From the perspective of social resources, the enterprises ought to maximize the utility of social support for customers. In other words, it is advised that online travel platform enterprises should foster a heart-touching atmosphere of social support for customers, and proactively guide customers in transforming their role and engaging in customer citizenship behavior. First, enterprises are advised to establish a system of customer relationship management to record the characteristics and preferences of customers, so as to form customer profiles and to accurately understand and meet both informational and emotional needs of customers. Second, enterprises ought to integrate the channels of communication with customers in a multidimensional manner. On the one hand, enterprises can provide real-time chat service for customers of the platform to ensure information resources and measures for front-line services, so as to promote communication between customers and front-line employees of the platform; On the other hand, enterprises can create forums on the platform or set up question-and-answer areas in the comments section of the platform to promote the communication between customers and customers, so as to promote the establishment of good social relations between customers and other customers. In addition, the enterprise should provide customers with sufficient information support and emotional support to solve problems. Sufficient informational support includes providing customers with solutions to the problems that may be encountered during traveling in advance, and developing customized and precise schemes based on the purpose of traveling for customers, such as the schemes of traveling around or daily commuting, and traveling between different places or in the same city. Furthermore, the enterprises can offer informational support by providing timely and effective information and suggestions when customers are encountered with difficulties during and after traveling. Sufficient emotional support refers to the measures taken by the front-line employees of enterprises, which include providing warm tips prior to the traveling of customers, and showing respect, care, sense of understanding and encouragement to customers when they encounter difficulties during and after traveling. Last but not least, enterprises are advised to establish a mechanism of encouraging the front-line employees or users to provide support of both informational and emotional value to customers. For example, enterprises can encourage the users of the platform to share travel experiences or stories, to participate in forums and to interact with users of the platform, so as to maximize their utility derived from social support.

(2) From the perspective of emotional experience, the enterprises ought to provide customers with optimal experience in consumption emotion as well as stable level of customer satisfaction. In other words, to guide the positive behavior of customers, enterprises should not only meet the needs of customers for acquiring optimal social support, but also mobilize their positive consumption emotion and generate a stable level of satisfaction. Therefore, first of all, while assisting customers to cope with the problems and providing customers with both informational and emotional support, the enterprises ought to foster positive emotional experiences for customers, such as excitement, pleasure and ease, etc. In terms of informational value support, enterprises are advised to formulate customized and specialized traveling schemes that are fun and interesting, or provide the traveling information service to customers through promotional activities, which itself may be small, but the goodwill is deep. Such moves can bring joyful surprises to customers, so as to generate positive emotions such as excitement and pleasure for customers. In terms of emotional value support, enterprises are advised to provide customers with a secure, reliable and comfortable traveling scheme, whereas the front-line employees of enterprises ought to pay attention to protecting customers’ privacy while assisting them to tackle with the problems, so as to ensure that customers feel delighted and relaxed. Secondly, the forum activities created by enterprises should be interesting, so as to stimulate customers’ pleasant experience. Finally, platform users are encouraged to share surprising, pleasant and satisfying travel content.

(3) The enterprises are required to focus on managing the positive emotions of customers. Compared with negative emotions, positive emotions can impose a more significant impact on customer citizenship behavior. Therefore, enterprises ought to focus on providing informational and emotional assistance for customers. Such moves will help mobilize the positive emotional experience of customers, thereby facilitating the customers’ engagement in citizenship behaviors.

### Research Deficiency and Prospect

If customers only require limited or no social support at all, the arbitrary provision of social support by enterprises could be counterproductive (Melrose et al., 2015). However, in this study, we have not taken into account the extent to which social support is optimal for guiding customers’ citizenship behaviors. Future studies can explore the negative impact imposed by false informational support or excessive emotional support provided by enterprises on customers and their behaviors. Social support and customer citizenship behavior are second-order constructs. This study only discusses the influence of the second-order variable of social support on the second-order variable of customer citizenship behavior. In the future, the influence of each dimension of social support on each dimension of customer citizenship behavior can be further explored. In this study, self-reported questionnaire data were used, so that the causal relationship between social support and consumer sentiment, as well as consumption emotion and customer satisfaction and customer citizenship behavior, could not be accurately tested. So the longitudinal study designs or experimental methods should be used to examine these relationships in future studies. Then transient sampling technique can be used to replace improbability convenience sampling method in order to measure consumer sentiment more accurately in future studies. Lastly, the selection of respondents is targeted at Chinese people, while online tourists tend to come from all over the world. In future studies, sample selection can be expanded to facilitate the application and promotion of research conclusions.

## Data Availability Statement

The original contributions presented in the study are included in the article/supplementary material, further inquiries can be directed to the corresponding author/s.

## Author Contributions

Y-MN wrote and revised the manuscript under the guidance of the supervisor CH. Both authors contributed to the article and approved the submitted version.

## Conflict of Interest

The authors declare that the research was conducted in the absence of any commercial or financial relationships that could be construed as a potential conflict of interest.

## Publisher’s Note

All claims expressed in this article are solely those of the authors and do not necessarily represent those of their affiliated organizations, or those of the publisher, the editors and the reviewers. Any product that may be evaluated in this article, or claim that may be made by its manufacturer, is not guaranteed or endorsed by the publisher.
